# Influence of the Lactotripeptides Isoleucine–Proline–Proline and Valine–Proline–Proline on Systolic Blood Pressure in Japanese Subjects: A Systematic Review and Meta-Analysis of Randomized Controlled Trials

**DOI:** 10.1371/journal.pone.0142235

**Published:** 2015-11-04

**Authors:** Aurelie Chanson-Rolle, François Aubin, Veronique Braesco, Toshimitsu Hamasaki, Masafumi Kitakaze

**Affiliations:** 1 VAB-nutrition, Clermont-Ferrand, France; 2 Venn Life Sciences, Paris, France; 3 Department of Advanced Medical Technology, National Cerebral and Cardiovascular Center, Osaka, Japan; 4 Department of Clinical Medicine and Development, National Cerebral and Cardiovascular Center, Osaka, Japan; University of Bologna, ITALY

## Abstract

**Background:**

The lactotripeptides isoleucine–proline–proline (IPP) and valine–proline–proline (VPP) have been shown to decrease systolic blood pressure (SBP) in several populations, but the size of the effect varies among studies. We performed a meta-analysis including all published studies to evaluate the SBP-lowering effect of IPP/VPP in Japanese subjects more comprehensively.

**Methods and Findings:**

Eligible randomized controlled trials were searched for within four bibliographic databases, including two Japanese ones. Eighteen studies (including a total of 1194 subjects) were included in the meta-analysis. A random effect model using the restricted maximum likelihood (REML) estimator was used for the analysis. The analysis showed that consumption of IPP/VPP induced a significant reduction in SBP as compared with placebo in Japanese subjects, with an estimated effect of -5.63 mm Hg (95% CI, -6.87 to -4.39, P<0.0001) and no evidence of publication bias. A significant heterogeneity between series was evident, which could be explained by a significant influence of the baseline blood pressure status of the subjects, the effect of IPP/VPP on SBP being stronger in hypertensive subjects (-8.35 mm Hg, P<0.0001) than in non-hypertensive subjects (-3.42mm Hg, P<0.0001). Furthermore, the effect of IPP/VPP on SBP remained significant when limiting the analysis to series that tested the usual doses of IPP/VPP consumed daily (below 5 mg/d), with estimated effects of -6.01 mm Hg in the overall population and -3.32 mm Hg in non-hypertensive subjects.

**Conclusions:**

Results from this meta-analysis show that IPP/VPP lactotripeptides can significantly reduce office SBP in Japanese subjects with or without overt hypertension, and for doses that can potentially be consumed as an everyday supplement. This suggests that these peptides could play a role in controlling blood pressure in Japanese subjects. The systematic review protocol was published on the PROSPERO register (CRD42014014322).

## Introduction

Hypertension is a major determinant of health and is likely to have an effect on medical economics worldwide, including in Asian countries such as Japan [1]. Lifestyle change measures such as salt restriction, moderation of alcohol consumption or regular physical exercise are recommended as the initial management of hypertension [2, 3]. In parallel, several randomized trials and meta-analyses have shown that some peptides derived from milk proteins, such as the lactotripeptides isoleucine–proline–proline (IPP) and valine–proline–proline (VPP), decrease systolic blood pressure (SBP) [4–6]. Because IPP and VPP were first isolated and identified as angiotensin-converting enzyme (ACE) inhibitory peptides [7] and were shown to exert antihypertensive effects after oral administration in spontaneously hypertensive rats [8, 9], both peptides had been thought to act through ACE inhibition [10]. However, this has not been clearly demonstrated, and other mechanisms might also be involved, such as production of vasodilative substances [11, 12] or an effect on sympathetic nervous activity [13].

Previous meta-analyses have consistently shown that IPP/VPP intake decreases SBP when compared with placebo, with estimated size effects of -4.8 mmHg (95% CI:, -6.0 to -3.7) [6], -3.73 mmHg (95% CI, -6.70 to -1.76) [4] or -2.95 mmHg (95% CI, -4.17 to -1.73) [14]. The size of the effect varies among studies, however, and at least part of the observed heterogeneity seems to be due to ethnicity, with a stronger effect observed in Asian subjects (-5.54 mmHg to -6.93 mmHg, depending on the meta-analysis) than in European subjects (-1.17 mmHg to -1.36 mmHg) [4, 14]. A recent meta-analysis of randomized controlled studies performed in European subjects confirmed that IPP/VPP was effective in moderately reducing SBP in this population, with an estimated effect of -1.28 mmHg (95%CI, -2.09 to -0.48) and no evidence of heterogeneity [15]. Several randomized controlled trials have been performed in Asian subjects, specifically Japanese subjects, but, for some, results have been published in journals written in the Japanese language only and not indexed in the main bibliographic databases. Available meta-analyses have not searched for such publications [4–6], and therefore no exhaustive meta-analysis has been performed to specifically assess the size of the effect in Asian subjects, specifically Japanese subjects. The objectives of the present meta-analysis were to estimate the size of the change in SBP after IPP/VPP consumption in Asian subjects, and to study the influence of the ingested dose and duration of IPP/VPP intake on this change, as well as the influence of age and baseline blood pressure (BP) status of the subjects.

## Material and Methods

The protocol of the systematic review and meta-analysis has been published on the PROSPERO register (http://www.crd.york.ac.uk/prospero/) under registration number CRD42014014322 (see also **S1 Protocol**). This study complies with the requirements of the Preferred Reporting Items for Systematic reviews and Meta-Analyses (PRISMA) statement [16] and the PRISMA checklist is available as supporting information (see **S1 Checklist**).

### Data sources and searches

Four bibliographic databases [MEDLINE (http://www.ncbi.nlm.nih.gov/pubmed), Cochrane Central Register of Controlled Trials (http://www.thecochranelibrary.com), J-STAGE (https://www.jstage.jst.go.jp/browse) and J Dream III (http://jdream3.com)] were systematically searched until October 1^st^, 2014, by using the following combination of keywords: [lactotripeptide* OR “dairy peptide*” OR (“Ile-Pro-Pro” AND “Val-Pro-Pro”) OR (“Isoleucyl-prolyl-proline” AND “valyl-prolyl-proline”) OR (“Valine-proline-proline” AND “isoleucine-proline-proline”) OR (“IPP” and “VPP”) OR “fermented milk” OR “milk fermented” OR “sour milk”] AND (hypertension OR "blood pressure"). J-STAGE and J Dream III are bibliographic databases for scientific publications written in the Japanese language. For the searches on the MEDLINE and Cochrane databases, the keywords “Asian OR Japan OR Japanese” were added to the search strategy. No restriction to language of publication was applied. In addition, the reference lists of included studies were searched for additional potentially eligible studies.

### Study selection

**Fig 1:**
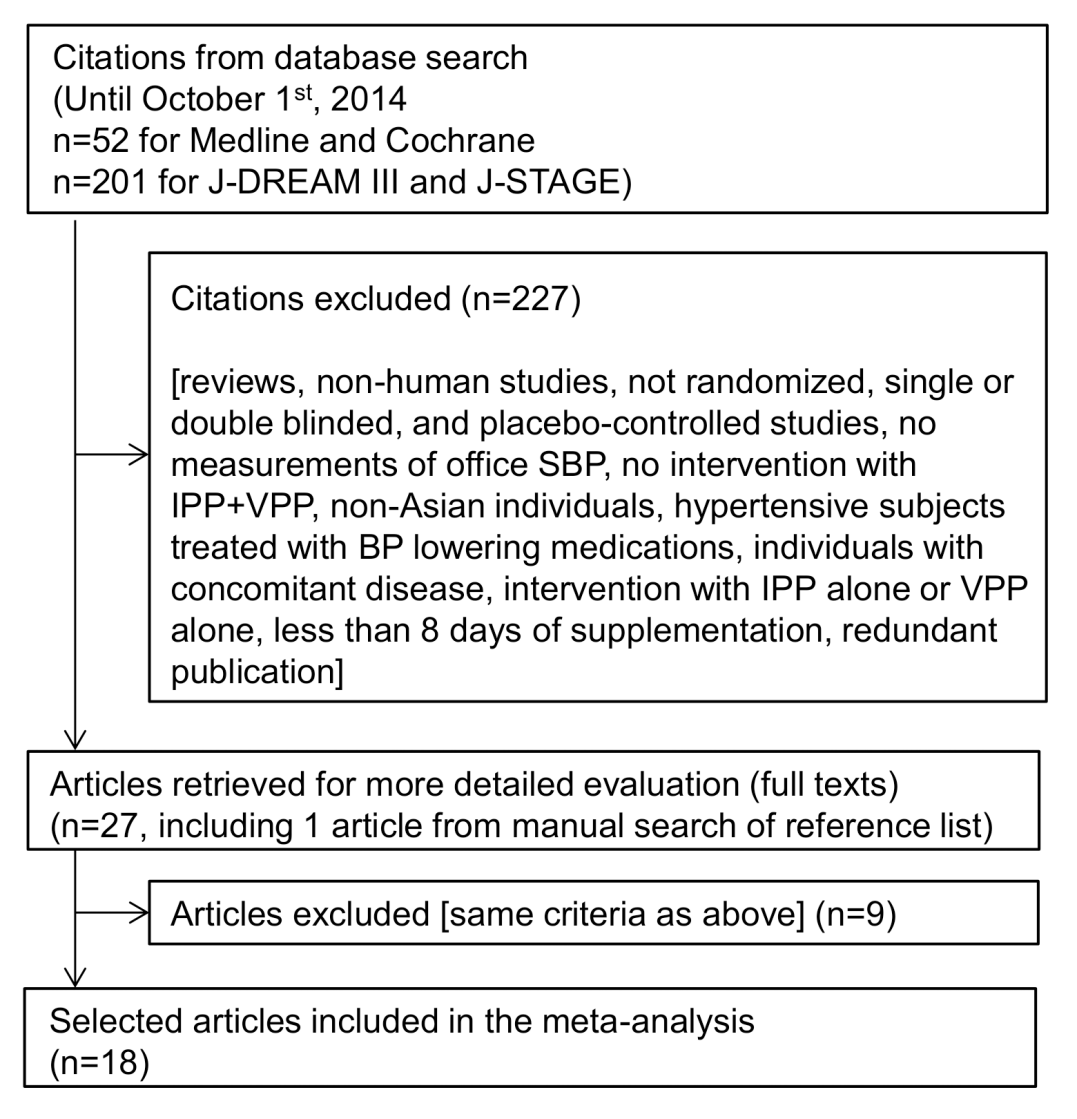
Flow diagram of study selection. The list of the 27 articles selected for full text evaluation is available in S1 Table, which also describes the outcome of the selection process for each article (including justification for exclusion). BP: blood pressure. IPP: isoleucine–proline–proline. SBP: systolic blood pressure. VPP: valine–proline–proline.

Three scientists with doctoral degrees (VB, TH, MK) independently screened the titles and abstracts of all retrieved publications for inclusion or exclusion. Full text articles were obtained when abstracts were potentially relevant and were reviewed independently; conflicting views were resolved by discussions. For publications in the Japanese language, English translations were obtained and used. Included studies were randomized placebo-controlled trials, single or double blinded, involving the consumption of IPP and VPP by Asian adult subjects for longer than 8 days, with office SBP measurements at baseline and one or more time points. Redundant publications, reviews, non-human studies or studies that were not randomized, not single or double blinded, or not placebo controlled, were excluded from the meta-analysis, as were studies in which no measurements of office SBP or no intervention with IPP+VPP were reported. In addition, studies performed in non-Asian individuals, in individuals with concomitant disease or in hypertensive subjects treated with BP-lowering medications, studies testing IPP alone or VPP alone or IPP+VPP for less than 8 days were also excluded (**Fig 1**).

### Data extraction

A predefined template was prepared for the collection of data. Extracted data were independently checked by a trained statistician (FA) and by a PhD-qualified researcher (ACR), with disagreements resolved through discussion. Authors from ten publications were contacted for further information. All responded and provided the level of information required. Extracted data for each trial included were: (i) characteristics of the study, including its design, duration and dose of IPP/VPP administration, country and year of publication; (ii) characteristics of participants (mean age, mean baseline BP status); (iii) primary outcome measure (change from baseline to final endpoint in office SBP), secondary outcome measure (change from baseline to 4 weeks in office SBP, change from baseline to final endpoint in office diastolic BP [DBP]), number of subjects for which study data were analyzed, mean effect and variability measures (standard deviation [SD] or standard error of the mean [SEM] or 95% confidence interval [CI]). Office measurement was chosen for the evaluation of SBP and DBP because it was the component of BP measured (in accord with good practice) in each study. Data on SBP and DBP changes and mean age were extracted separately for each type of subject, i.e., for subjects with normal BP (normotensive [NT]), high-normal BP (pre-hypertensive [PHT]) and high BP (hypertensive [HT]), when corresponding data were available. Quality of individual studies was assessed by using the Jadad score [17].

### Statistical analysis

The outcome measure was the mean difference between groups receiving IPP/VPP and those receiving placebo in the change from baseline to final endpoint in office SBP (primary outcome) and office DBP (secondary outcome). Both fixed and random effects were estimated to calculate the mean pooled effect of IPP/VPP and its 95% CI. Because heterogeneity was suspected based on the available literature [4], the random effect model meta-analysis, using the REML (REstricted Maximum Likelihood) estimator [18, 19], was considered as the primary analysis. Studies were weighted according to the inverse of their variance. Between-studies heterogeneity was quantified by computing the standard tau², I² and H² statistics and by computing the Cochran’s Q test statistic [20]. The potential for publication bias was explored by producing a funnel plot (plotting SE of effect versus estimate of effect-size for each study) and by computing the Kendall’s rank correlation test statistic (Kendall’s tau) between the standardized effect-size and the SE values of the effect, as proposed by Begg and Mazumdar (1994) [21].

Heterogeneity of the effect of IPP/VPP on SBP was explored by conducting adjusted meta-analyses, meta-regressions and subgroup analyses. The influence of the following trial characteristics was investigated: type of subjects (on the basis of their baseline BP status: NT, PHT or HT), mean age of the subjects, daily dose, and duration of IPP/VPP intake. Subgroup meta-analyses were performed on office SBP changes after 4 weeks of supplementation with IPP/VPP and on office SBP changes at final endpoint within series testing IPP/VPP doses lower than 5 mg/d, which correspond to the doses that can potentially be consumed as an everyday supplement. Subgroup meta-analyses were also performed on office SBP changes at final endpoint within NT, PHT and HT subjects, separately, and for NT and PHT subjects considered together (subgroup of non-HT subjects). The influence of each individual study on the overall results was analyzed by omitting one study at a time. Influence effects were also computed, using studentized residuals, Cook’s distance, and hat value.

All analyses were preplanned and described in a statistical analysis plan, except for the subgroup analyses that were used to explore interactions between covariates. We used the Metafor package [19] version 1.4–0 under R version 2.15.2 (the R Foundation for Statistical Computing, Vienna, Austria) for all the computations in our statistical analysis of data.

## Results

### Characteristics of included studies

**Fig 2:**
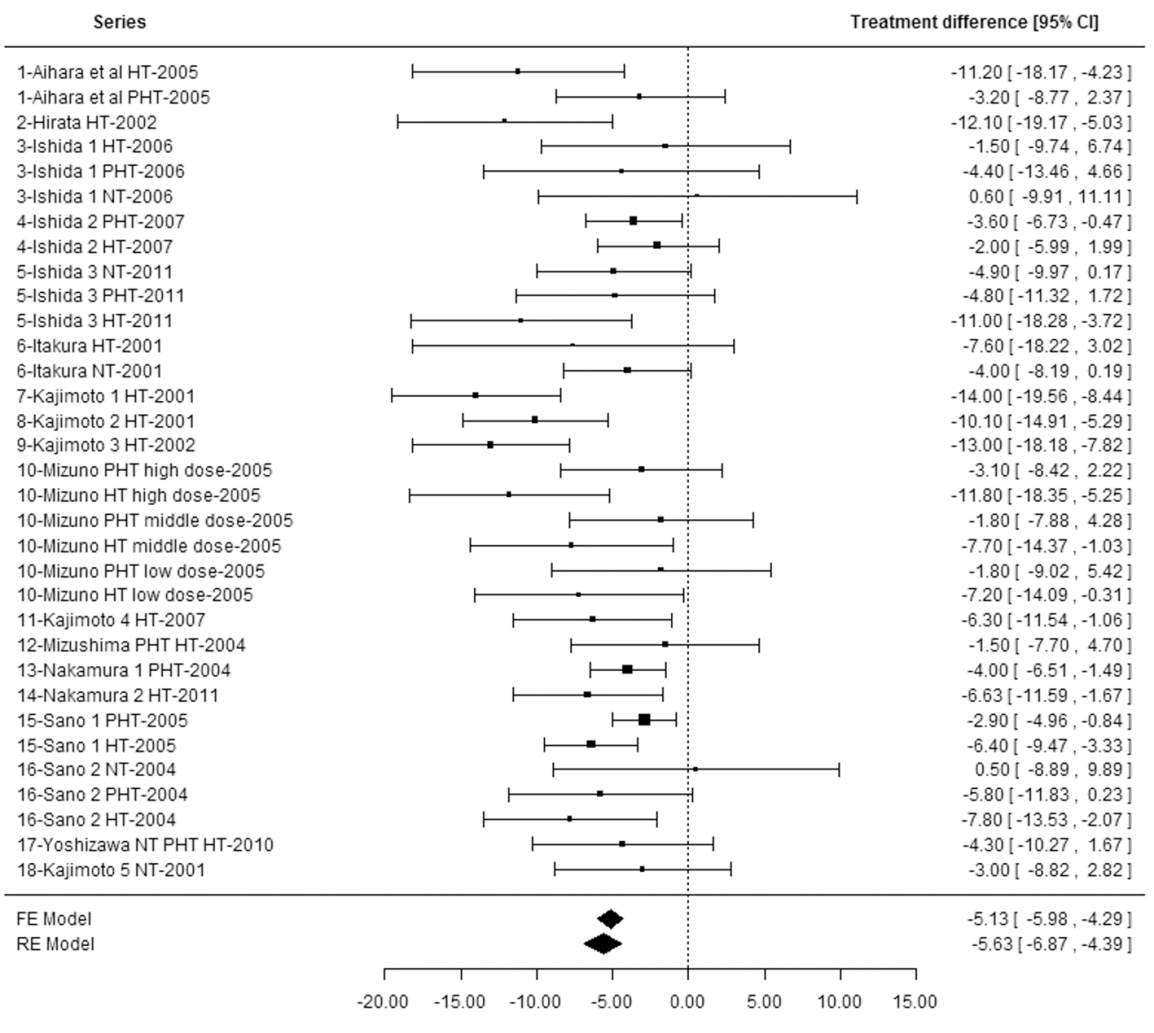
Forest plot of treatment effects of isoleucine–proline–proline/valine–proline–proline in the meta-analysis of 33 series of findings of its effect on systolic blood pressure in Japanese subjects. FE: fixed effect. HT: hypertensive. NT: normotensive. PHT: pre-hypertensive. RE: random effect. SBP: systolic blood pressure.

A total of 253 publications were identified (including 201 from the Japanese databases). On the basis of the criteria described above, 236 publications were discarded (Fig 1) and 17 studies were included [22–38]. One additional study [39] was identified by hand search from the reference list of selected papers, totaling 18 included studies. All of the studies were performed in Japanese subjects, and were published as full papers. Seven studies were published in English in international and peer-reviewed journals [22, 25, 32–34, 37, 38], and 11 studies were published in the Japanese language [23, 24, 26–31, 35, 36, 39] in Japanese journals that were all peer-reviewed but one (corresponding to two publications: [29, 39]). English translations were obtained from all 11 Japanese studies. Thirty-three series, defined by type of subjects (NT, PHT and/or HT) and dose of IPP/VPP, were analyzed from the 18 studies included (**Table 1**). More precisely, 12 studies (20 series) tested doses of IPP/VPP that were lower than 5 mg/d, which correspond to doses that could potentially be consumed as an everyday supplement. In the six remaining studies (13 series), tested doses were higher than the “usual” doses and comprised between 6.7 and 17.1 mg/d, because those studies were performed to examine the safety of IPP/VPP at doses greatly exceeding the usual daily doses. Overall, the 33 reported series that were analyzed included 1194 treatment periods (633 with IPP/VPP and 561 with placebo) in 1194 subjects. NT, PHT and HT subjects were studied separately in five, 10 and 16 series, respectively (corresponding to a total number of 114, 419 and 597 subjects, respectively). The three types of subjects were studied together in one series (22 subjects from the Yoshizawa et al 2010 study [38]), and HT and PHT subjects were considered together in another series (45 subjects from the Mizushima et al 2004 study [33]). All of the included studies were randomized controlled trials with parallel design and double blinded, except two, which were single-blinded [32, 38] (Table 1). All data used for the meta-analysis were found in the publications or obtained directly from the authors, which, together with estimation of study quality (JADAD score equal or higher than 3 except for one study [38]), indicate that the risk of bias within studies was minimized. For the primary outcome (change from baseline to final endpoint in office SBP), the mean difference between IPP/VPP and placebo varied among individual studies from -14.0 (± 2.8 SE) mm Hg in favor of IPP/VPP to +0.6 (± 5.4 SE) in favor of placebo (**Table 2** and **Fig 2**). Data related to DBP are provided in **S2 Table**.

**Table 1:** Characteristics of the 18 studies included in the meta-analysis of randomized controlled trials of the effect of isoleucine–proline–proline and valine–proline–proline on systolic blood pressure in Japanese subjects.

			Intervention				Population		
Study #	Reference	Jadad score	IPP/VPP dose (mg/d)	Duration(weeks)	Design	Country	Type of subjects (BP status)	n analyzed	Mean age (y)
1	Aihara 2005 [22]	4	13	4	D	Japan	HT	40	51.7
1	Aihara 2005 [22]	4	13	4	D	Japan	PHT	40	51.4
2	Hirata 2002 [39]	3	4.3	8	D	Japan	HT	32	50.7
3	Ishida 1–2006 [23]	4	15.7	4	D	Japan	HT	18	55.5
3	Ishida 1–2006 [23]	4	15.7	4	D	Japan	PHT	18	51.2
3	Ishida 1–2006 [23]	4	15.7	4	D	Japan	NT	18	48.9
4	Ishida 2–2007 [24]	4	3.6	12	D	Japan	PHT	71	50.3
4	Ishida 2–2007 [24]	4	3.6	12	D	Japan	HT	40	51.8
5	Ishida 3–2011 [25]	4	17.1	4	D	Japan	NT	16	44.2
5	Ishida 3–2011 [25]	4	17.1	4	D	Japan	PHT	16	49.6
5	Ishida 3–2011 [25]	4	17.1	4	D	Japan	HT	16	54.2
6	Itakura 2001 [26]	3	2.6	8	D	Japan	HT	18	54.5
6	Itakura 2001 [26]	3	2.6	8	D	Japan	NT	26	36.0
7	Kajimoto 1–2001 [31]	4	4.1	8	D	Japan	HT	30	52.0
8	Kajimoto 2–2001 [27]	4	4.2	8	D	Japan	HT	81	45.9
9	Kajimoto 3–2002 [30]	4	3.8	8	D	Japan	HT	64	50.0
10	Mizuno 2005 [32]	3	3.6	6	S	Japan	PHT	24	42.8
10	Mizuno 2005 [32]	3	3.6	6	S	Japan	HT	41	45.9
10	Mizuno 2005 [32]	3	2.5	6	S	Japan	PHT	24	46.4
10	Mizuno 2005 [32]	3	2.5	6	S	Japan	HT	41	43.9
10	Mizuno 2005 [32]	3	1.8	6	S	Japan	PHT	24	45.0
10	Mizuno 2005 [32]	3	1.8	6	S	Japan	HT	41	43.0
11	Kajimoto 4–2007 [29]	4	3.55	8	D	Japan	HT	49	56.1
12	Mizushima 2004 [33]	4	3.1	4	D	Japan	PHT & HT	42	46.3
13	Nakamura 1–2004 [35]	4	3.7	12	D	Japan	PHT	106	38.5
14	Nakamura 2–2011 [34]	5	3.4	8	D	Japan	HT	70	57.8
15	Sano 1–2005 [37]	4	3.1	12	D	Japan	PHT	104	49.0
15	Sano 1–2005 [37]	4	3.1	12	D	Japan	HT	40	56.0
16	Sano 2–2004 [36]	4	9.2	4	D	Japan	NT	11	44.6
16	Sano 2–2004 [36]	4	9.2	4	D	Japan	PHT	16	45.5
16	Sano 2–2004 [36]	4	9.2	4	D	Japan	HT	16	49.7
17	Yoshizawa 2010 [38]	2	6.7	8	S	Japan	NT, PHT & HT	22	57.5
18	Kajimoto 5–2001 [28]	4	12.4	2	D	Japan	NT	43	29.7

BP: blood pressure. D: double-blinded. IPP: isoleucine–proline–proline. HT: hypertensive. NT: normotensive. n: number of subjects. PHT: pre-hypertensive. S: single-blinded. VPP: valine–proline–proline. y: years.

**Table 2:** Effect of isoleucine–proline–proline and valine–proline–proline on systolic blood pressure at final endpoint.

Study #	Series number	Study reference	Type of subjects (BP status)	IPP/VPP dose (mg/d)		Treated group			Placebo group		Effect size	
					n	Change in SBP (mmHg)		n	Change in SBP (mmHg)		Mean difference between groups	SE
						mean	SD		mean	SD		
1	101	Aihara 2005 [22]	HT	13.0	20	NA	NA	20	NA	NA	-11.2	3.6
1	102	Aihara 2005 [22]	PHT	13.0	20	NA	NA	20	NA	NA	-3.2	2.8
2	201	Hirata 2002 [39]	HT	4.3	16	-14.5	9.9	16	-2.4	10.5	-12.1	3.6
3	301	Ishida 1–2006 [23]	HT	15.7	9	-0.9	7.0	9	0.6	10.5	-1.5	4.2
3	302	Ishida 1–2006 [23]	PHT	15.7	9	-8.3	10.0	9	-3.9	9.6	-4.4	4.6
3	303	Ishida 1–2006 [23]	NT	15.7	9	-0.7	8.6	9	-1.3	13.6	0.6	5.4
4	401	Ishida 2–2007 [24]	PHT	3.6	35	-6.8	8.4	36	-3.2	7.5	-3.6	1.6
4	402	Ishida 2–2007 [24]	HT	3.6	20	-9.8	8.1	20	-7.8	7.1	-2.0	2.0
5	501	Ishida 3–2011 [25]	NT	17.1	8	-2.5	5.6	8	2.4	4.7	-4.9	2.6
5	502	Ishida 3–2011 [25]	PHT	17.1	8	-6.7	8.1	8	-1.9	4.8	-4.8	3.3
5	503	Ishida 3–2011 [25]	HT	17.1	8	-14.9	6.7	8	-3.9	8.1	-11.0	3.7
6	601	Itakura 2001 [26]	HT	2.6	9	-12.1	14.1	9	-4.4	8.1	-7.6	5.4
6	602	Itakura 2001 [26]	NT	2.6	13	-3.3	5.3	13	0.7	5.6	-4.0	2.1
7	701	Kajimoto 1–2001 [31]	HT	4.1	15	-13.7	10.1	15	0.3	4.3	-14.0	2.8
8	801	Kajimoto 2–2001 [27]	HT	4.2	42	-12.4	10.9	39	-2.3	11.2	-10.1	2.5
9	901	Kajimoto 3–2002 [30]	HT	3.8	31	-13.9	11.4	33	-0.9	9.7	-13.0	2.6
10	1001	Mizuno 2005 [32]	PHT	3.6	12	-2.8	5.8	12	0.3	7.4	-3.1	2.7
10	1002	Mizuno 2005 [32]	HT	3.6	21	-13.0	10.4	20	-1.2	11.0	-11.8	3.3
10	1003	Mizuno 2005 [32]	PHT	2.5	12	-1.5	7.8	12	0.3	7.4	-1.8	3.1
10	1004	Mizuno 2005 [32]	HT	2.5	21	-8.9	10.8	20	-1.2	11.0	-7.7	3.4
10	1005	Mizuno 2005 [32]	PHT	1.8	12	-1.5	10.4	12	0.3	7.4	-1.8	3.7
10	1006	Mizuno 2005 [32]	HT	1.8	21	-8.4	11.5	20	-1.2	11.0	-7.2	3.5
11	1101	Kajimoto 4–2007 [29]	HT	3.6	25	-4.3	8.8	24	2.0	9.9	-6.3	2.7
12	1201	Mizushima 2004 [33]	PHT & HT	3.1	22	-5.2	10.8	20	-3.7	9.6	-1.5	3.2
13	1301	Nakamura 1–2004 [35]	PHT	3.7	53	-6.1	5.7	53	-2.1	8.4	-4.0	1.3
14	1401	Nakamura 2–2011 [34]	HT	3.4	35	-10.5	11.5	35	-3.9	9.6	-6.6	2.5
15	1501	Sano 1–2005 [37]	PHT	3.1	52	-4.6	6.2	52	-1.7	6.2	-2.9	1.1
15	1502	Sano 1–2005 [37]	HT	3.1	20	-9.5	7.5	20	-3.1	4.6	-6.4	1.6
16	1601	Sano 2–2004 [36]	NT	9.2	6	0.8	4.4	5	0.3	10.8	0.5	4.8
16	1602	Sano 2–2004 [36]	PHT	9.2	8	-4.4	6.9	8	1.4	5.3	-5.8	3.1
16	1603	Sano 2–2004 [36]	HT	9.2	8	-9.5	5.1	8	-1.7	6.5	-7.8	2.9
17	1701	Yoshizawa 2010 [38]	NT, PHT & HT	6.7	12	-6.1	8.2	10	-1.8	5.5	-4.3	3.0
18	1801	Kajimoto 5–2001 [28]	NT	12.4	21	-3.9	7.9	22	-0.9	11.2	-3.0	3.0

BP: blood pressure. IPP: isoleucine–proline–proline. HT: hypertensive. n: number of subjects. NA: not available. NT: normotensive. PHT: pre-hypertensive. SBP: systolic blood pressure. VPP: valine–proline–proline.

### Effect of isoleucine–proline–proline and valine–proline–proline on blood pressure

The results of the primary meta-analysis (SBP changes at final endpoint) performed with the random effect model showed a statistically significant greater effect of IPP/VPP over placebo on SBP in Japanese subjects, of a magnitude of -5.63 mm Hg (95% CI, -6.87 to -4.39, P<0.0001) (**Fig 2**). The results with the fixed effect model were consistent [effect estimate of -5.13 mm Hg (95% CI, -5.98 to -4.29)]. The estimate of the effect of IPP/VPP on DBP was smaller but significant [-2.58 mmHg (95% CI, -3.44 to -1.72), P<0.0001] (**S1 Fig**).

Heterogeneity between series (I^2^ = 44.4%, tau^2^ = 5.05) was significant for SBP (Q = 56.1, P = 0.0053), but not for DBP (I^2^ = 25.4%, tau^2^ = 1.5 and Q = 40.7, P = 0.14).

### Exploration of heterogeneity and subgroup meta-analyses

**Fig 3:**
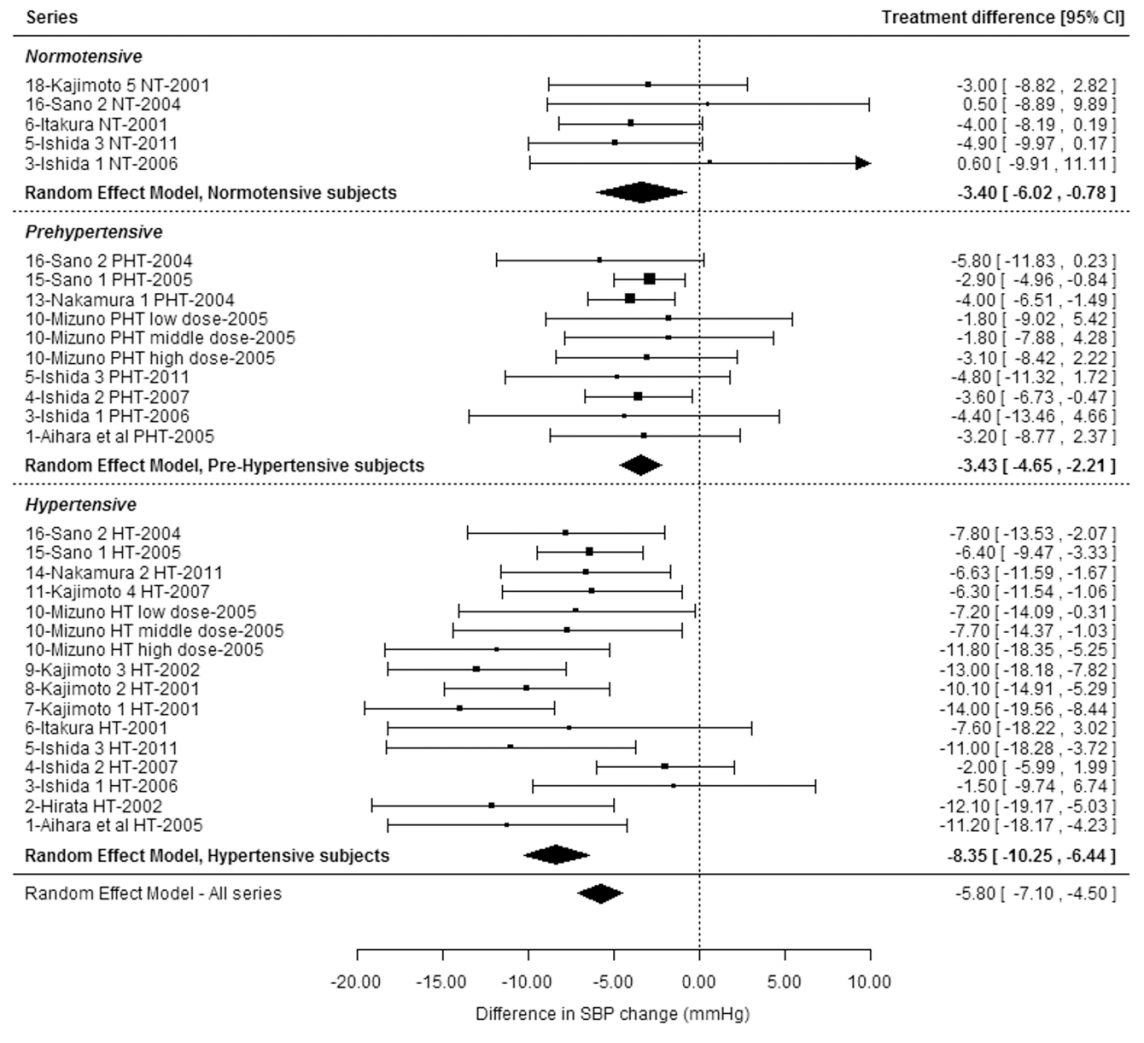
Forest plot of treatment effects of isoleucine–proline–proline/valine–proline–proline on systolic blood pressure in the subgroup analysis according to the baseline blood pressure status of the subject. Data on SBP changes were available separately for NT, PHT and/or HT subjects for all studies except two, which were therefore excluded from this subgroup analysis (Mizushima et al 2004 [33] and Yoshizawa et al 2010 [38]). HT: hypertensive. NT: normotensive. PHT: pre-hypertensive. SBP: systolic blood pressure.

Exploration of heterogeneity was performed through adjusted meta-analyses, meta-regressions and subgroup analyses of SBP data. It provides evidence for the following: (i) A significant influence of the baseline BP status of the subjects (P < 0.0001), with subgroup analyses showing a higher effect-size in HT subjects [-8.35 mm Hg (95%CI, -10.25 to -6.44)] than in NT subjects [-3.40 mm Hg (95%CI, -6.02 to -0.78)] and PHT subjects [-3.43 mm Hg (95%CI, -4.65 to -2.21)], although the IPP/VPP effect on SBP was significant in all three groups of subjects (P < 0.0001, P = 0.0109 and P < 0.0001, respectively) (**Fig 3**). The effect was also significant (P < 0.0001) in non-HT subjects (i.e., in NT and PHT subjects pooled together), with an effect-size of -3.42 mm Hg (95%CI, -4.53 to -2.32). Interestingly, when adjusting the meta-analysis on type of subjects, the estimates of heterogeneity (I^2^ = 10.5%, tau^2^ = 0.76) were largely reduced when compared with the unadjusted analysis and the Q test became non-significant (Q = 29.1, P = 0.41). (ii) A non-significant influence of the daily dose of IPP/VPP was observed, when it was analyzed as a continuous variable (P = 0.78) or as a binary variable (below vs above 5 mg/d, i.e., “usual” vs higher than usual doses, respectively) (P = 0.53). A potential confounding effect between dose and duration was apparent, however: as shown in Table 1, most studies (five out of six) that tested doses of IPP/VPP above 5 mg/d were of short duration (2 or 4 weeks). Therefore, in order to suppress the potential confounding effect of duration, we performed a subgroup analysis of the effect of IPP/VPP on SBP after 4 weeks of supplementation, which was the time-point with the greatest number of series with available results (data available for 24 series). Daily dose of IPP/VPP still did not have a significant influence on the effect of IPP/VPP on SBP in this subgroup analysis (P = 0.26 and 0.34 when dose was analyzed as a continuous or binary variable, respectively).

In the subgroup analysis on the 20 series that tested doses lower than 5 mg/d, the IPP/VPP effect on SBP (final endpoint) remained significant (P < 0.0001), with a decrease of -6.01 mm Hg in comparison with placebo (95%CI, -7.70 to -4.32) and persistence of a significant heterogeneity (Q = 45.5, P = 0.0006). There was also evidence of a significant influence of the baseline BP status of the subjects in this subgroup analysis (P = 0.0011), with a larger effect-size in HT subjects [-8.44 mm Hg (95%CI, -10.68 to -6.19)] than in PHT subjects [-3.25 mm Hg (95%CI, -4.57 to -1.93)] or in the single series of NT subjects [-4.00 mm Hg (95%CI, -8.19 to 0.19) [26]]. When NT and PHT subjects were pooled together, the estimated effect-size was -3.32 mm Hg (95%CI, -4.57 to -2.06), showing a significant (P < 0.0001) effect of doses of IPP/VPP lower than 5 mg/d in the subgroup of non-HT subjects.

No significant influence of duration of intake (P = 0.71) or mean age (P = 0.18) of the subjects was seen on the estimate of the reduction in SBP.

For DBP, only the influence of the baseline BP status of the subjects on the effect of IPP/VPP was explored, with once again a higher effect-size in HT subjects (-3.63 mm Hg, P < 0.0001) than in PHT subjects (-1.71 mm Hg, P = 0.0177) and NT subjects (-1.18 mm Hg, not statistically significant) (**S2 Fig**). The effect of IPP/VPP on DBP in non-HT subjects (NT and PHT subjects pooled together) was significant (P = 0.0067), with an estimated effect-size of -1.53 mm Hg.

### Influence of individual studies and publication bias

The effect of leaving each series out and influence diagnostics showed that no series had a strong influence likely to have biased the results, the meta-analyses consistently showing a statistically significant effect (P < 0.0001) of IPP/VPP on SBP whichever series was omitted. Two series were found to have the highest contribution to heterogeneity, however, namely series #701 and #901 (with residual Q of 46.1 and 47.0 upon removal of each study, respectively). Both were series of HT subjects and were those presenting the two highest observed effect-sizes (-14.0 and -13.0 mmHg, respectively) (see Table 2).

**Fig 4:**
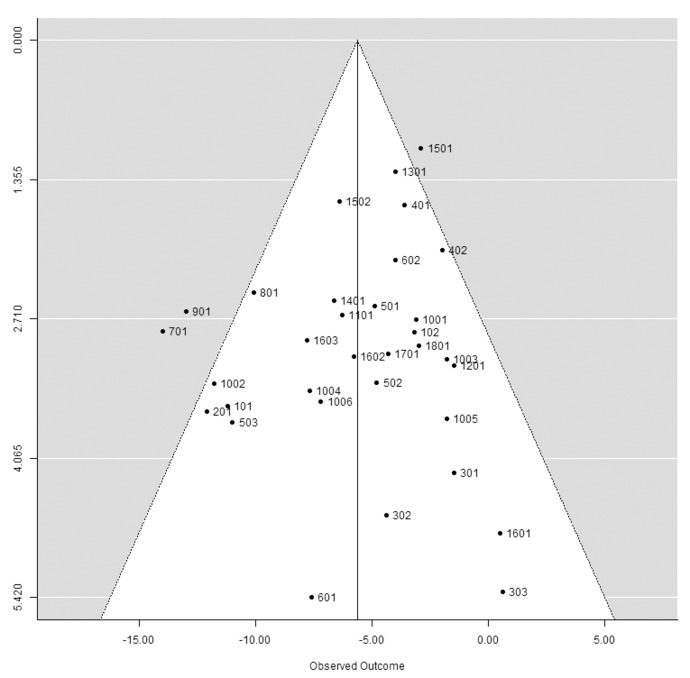
Funnel plot used to explore the potential for publication bias in the meta-analysis of 33 series for the effect of isoleucine–proline–proline/valine–proline–proline on systolic blood pressure in Japanese subjects. Series numbers are those indicated in Table 2. IPP: isoleucine–proline–proline. SBP: systolic blood pressure. VPP: valine–proline–proline.

There was no publication bias on the basis of visual inspection of the funnel plot, with no evidence of asymmetry (**Fig 4**), or on the basis of the non-significant Kendall’s Tau statistic (Kendall’s Tau = -0.0379, P = 0.7702). The same lack of publication bias was true for DBP (**S3 Fig**).

## Discussion

The meta-analysis of the 18 identified randomized controlled trials performed in Asian subjects, all Japanese, and performed with the preplanned random effect model, showed that consumption of IPP/VPP induced a significant reduction in SBP as compared with placebo, with an estimated effect of -5.63 mm Hg (95% CI, -6.87 to -4.39, P < 0.0001) and no evidence of publication bias. A significant heterogeneity between series was evident, which was due to differences in magnitude of the effect. All studies showed either an effect favoring IPP/VPP or absence of effect and no studies showed an effect in favor of placebo. At least part of the heterogeneity could be explained by a significant influence of the baseline BP status of the subjects, with the effect of IPP/VPP on SBP being stronger in HT subjects (-8.35 mm Hg) than in non-HT subjects (-3.42 mm Hg). Interestingly, however, the SBP-reducing effect of IPP/VPP remained significant in non-HT subjects. Furthermore, subgroup analysis showed that the effect of IPP/VPP on SBP remained significant when limiting the analysis to the 20 series that tested usual daily doses (below 5 mg/d) of IPP/VPP (-6.01 mm Hg). This was also true in the subgroup of non-HT subjects, with an estimated effect-size of -3.32 mm Hg. This observation confirmed the capability of IPP/VPP to reduce SBP in HT and non-HT Japanese subjects at doses likely to be consumed as an everyday supplement.

Cohort studies performed in Asian, including Japanese, populations have shown significant associations between SBP and mortality and between SBP and risk of cardiovascular diseases, with, for the latter, a potential benefit of lowering SBP down to levels of at least 115 mm Hg [40, 41]. Therefore, the moderate but significant SBP-reducing effect of IPP/VPP observed in non-HT subjects may be useful at the population level, in particular for the prevention of cardiovascular events. Furthermore, several recommended and efficient lifestyle changes, such as moderate alcohol consumption or dietary sodium limitations, translate into SBP reductions of a similar or even lower magnitude [42–44]. Therefore, results from our meta-analysis, which is to our knowledge the first one to have pooled data from all available studies performed in Japanese populations, including those published in Japanese journals, confirm that consumption of IPP/VPP can be useful for helping to maintain a normal SBP or to better control SBP in Asian populations, including in subjects without overt hypertension.

The SBP-reducing effect of IPP/VPP estimated in our meta-analysis (-5.63 mmHg) is higher than the one obtained in a recent meta-analysis that included European subjects only (-1.28 mmHg) [15]. This observation confirms that the efficacy of IPP/VPP may be influenced by ethnicity, with a stronger effect observed in Asian subjects than in European subjects, as previously suggested [4, 14]. Reasons for this differential effect are not currently understood, but it may be related to different pharmacokinetics of IPP/VPP in Asians as compared to Europeans, or to differences in environmental factors including diet [4, 14].

Exploratory analyses showed that the daily dose had no significant influence on the effect of IPP/VPP on SBP. Although there could be a confounding effect between dose and duration (all studies with high doses, but one, were of short duration), this was unlikely to have affected this conclusion because the influence of the dose remained non-significant in the subgroup analysis performed on SBP changes after 4 weeks of supplementation with IPP/VPP (the duration for which the largest number of data were available). Similarly, age was not shown to exert a significant influence on the IPP/VPP effect on SBP, despite a relatively wide range of age across included studies (mean age from 29.7 y to 57.8 y). No influence of duration of IPP/VPP consumption was demonstrated either; however, the effect of duration of intake may have been confounded by the fact that longer-term studies were predominantly performed in PHT subjects (78% of the subjects with 12-week data), in whom the IPP/VPP effect was shown to be smaller. Moreover, IPP/VPP may be produced by fermentation or enzymatic hydrolysis. Although the end product is the same, some data in the literature [45, 14] suggest that the mode of preparation may influence the efficacy of the compounds. Data from our meta-analysis do not support this hypothesis in Japanese subjects, however, since we did not find a significant influence of the type of IPP/VPP ingredient (fermented vs enzymatic) on the SBP-reducing effect, the effect remaining significant with both types of compounds (data not shown). Finally, few of the included trials assessed other parameters than office BP. Although twenty-four-hour ambulatory BP was not assessed in any trials, brachial-ankle pulse wave velocity and flow-mediated dilatation were measured in two trials [34, 38], both showing a significant benefit of IPP/VPP consumption as compared to placebo.

A strength of our meta-analysis lies in the fact that we specifically searched for Japanese databases in order to identify any relevant studies published in Japanese journals that are not indexed in the MEDLINE or Cochrane databases. This allowed inclusion of a larger number of relevant studies than the previously published meta-analyses (e.g., [4]), and better assessment of the size of the effect of IPP/VPP on SBP in Asian subjects. Another strength of our study is that BP data were extracted for NT, PHT and HT subjects separately, which improved characterization of the magnitude of the effect of IPP/VPP according to the subjects’ BP status. Our meta-analysis also displayed several indicators of robustness, including no evidence of publication bias and no strong influence of one single study on the overall result. Furthermore, the risk of bias within studies was minimized by the fact that all studies included were randomized placebo-controlled trials that were all double blinded, except two which were single-blinded, and that all data used for the meta-analysis were found in the publications or obtained directly from the authors. Our meta-analysis also has some limitations, however, which may affect the interpretation of the results and should therefore be acknowledged. Although the total number of included studies and subjects was substantial, conclusions from some of the subgroup analyses may be considered with more caution given the smaller number of available data. For example, the sub-analysis on the NT subjects included five series for a total number of 114 subjects. Another limitation is that the considered studies did not include data about the spontaneous dietary intakes of IPP/VPP by the enrolled subjects at baseline and during the trials. However, this should have had a limited impact since subjects were instructed to not change their dietary habits throughout the trials. Moreover, despite the lack of evidence for any publication bias in the studies included in our meta-analysis, some relevant studies may still not have been published. Finally, two of the Japanese studies included in our meta-analysis were published in a non-peer-reviewed journal [29, 39], but their individual quality was considered as appropriate (JADAD score of 3 and 4, respectively).

In conclusion, results from our meta-analysis show that the milk-derived peptides IPP and VPP can significantly reduce office SBP in Japanese subjects, with a statistically and clinically significant effect-size that may lead to a reduction in the risk of cardiovascular diseases at the population level [46]. Furthermore, the SBP reduction was observed in hypertensive subjects as well as in subjects without overt hypertension (NT and PHT subjects), and for a wide range of age, duration of intake and IPP/VPP doses, including doses that can potentially be consumed as an everyday supplement. This suggests that in Japanese subjects, IPP and VPP could play a role in the treatment of hypertension in hypertensive subjects, but also in the prevention of hypertension in subjects with normal or high-normal BP, in which consumption of foods containing IPP/VPP could help to maintain a normal BP or to better control BP. Additional studies are still required, however, to evaluate the antihypertensive efficacy of the IPP and VPP peptides further, particularly in subjects with normal or high-normal BP.

## Supporting Information

S1 Checklist PRISMA checklist for the Reporting of Meta-analyses of Randomized Controlled Trials. (DOC)

S1 Fig Forest plot of treatment effects of isoleucine–proline–proline/valine–proline–proline in the meta-analysis of 33 series of findings of its effect on diastolic blood pressure in Japanese subjects. DBP: diastolic blood pressure. FE: fixed effect. HT: hypertensive. NT: normotensive. PHT: pre-hypertensive. RE: random effect.
(TIFF)

S2 Fig Forest plot of treatment effects of isoleucine–proline–proline/valine–proline–proline) on diastolic blood pressure in the subgroup analysis according to the baseline blood pressure status of the subject. Data on SBP changes were available separately for NT, PHT and/or HT subjects for all studies except two, which were therefore excluded from this subgroup analysis (Mizushima et al 2004 [33] and Yoshizawa et al 2010 [38]). DBP: diastolic blood pressure. HT: hypertensive. NT: normotensive. PHT: pre-hypertensive.
(TIFF)

S3 Fig Funnel plot used to explore the potential for publication bias in the meta-analysis of 33 series for the effect of isoleucine–proline–proline/valine–proline–proline on diastolic blood pressure in Japanese subjects. Kendall’s Tau statistic: Kendall’s Tau = -0.1591, P = 0.2001. Series numbers are those indicated in S2 Table. DBP: diastolic blood pressure. IPP: isoleucine–proline–proline. VPP: valine–proline–proline.
(TIFF)

S1 Protocol Protocol of the systematic review as published on the PROSPERO register. (PDF)

S1 Table List of the 27 articles selected for full text evaluation. The outcome of the selection process is indicated for each article (with justification for exclusion).
(XLSX)

S2 Table Effect of isoleucine–proline–proline and valine–proline–proline on diastolic blood pressure at final endpoint. BP: blood pressure. DBP: diastolic blood pressure. IPP: isoleucine–proline–proline. HT: hypertensive. n: number of subjects. NA: not available. NT: normotensive. PHT: pre-hypertensive. VPP: valine–proline–proline.
(DOCX)

## Abbreviations

ACE: angiotensin-converting enzyme
BP: blood pressure
CI: confidence interval
DBP: diastolic blood pressure
FE: fixed effect
HT: hypertensive
IPP: isoleucine–proline–proline
NT: normotensive
PHT: pre-hypertensive
PRISMA: Preferred Reporting Items for Systematic reviews and Meta-analyses
RE: random effect
REML: restricted maximum likelihood
SBP: systolic blood pressure
SD: standard deviation
SE: standard error
SEM: standard error of the mean
VPP: valine–proline–proline
