# Supplementary figures and images for: Influence of the Lactotripeptides Isoleucine–Proline–Proline and Valine–Proline–Proline on Systolic Blood Pressure in Japanese Subjects: A Systematic Review and Meta-Analysis of Randomized Controlled Trials
Source: PLoS One. 2015 Nov 4;10(11):e0142235. doi: 10.1371/journal.pone.0142235 (PMC4633157; doi:10.1371/journal.pone.0142235)

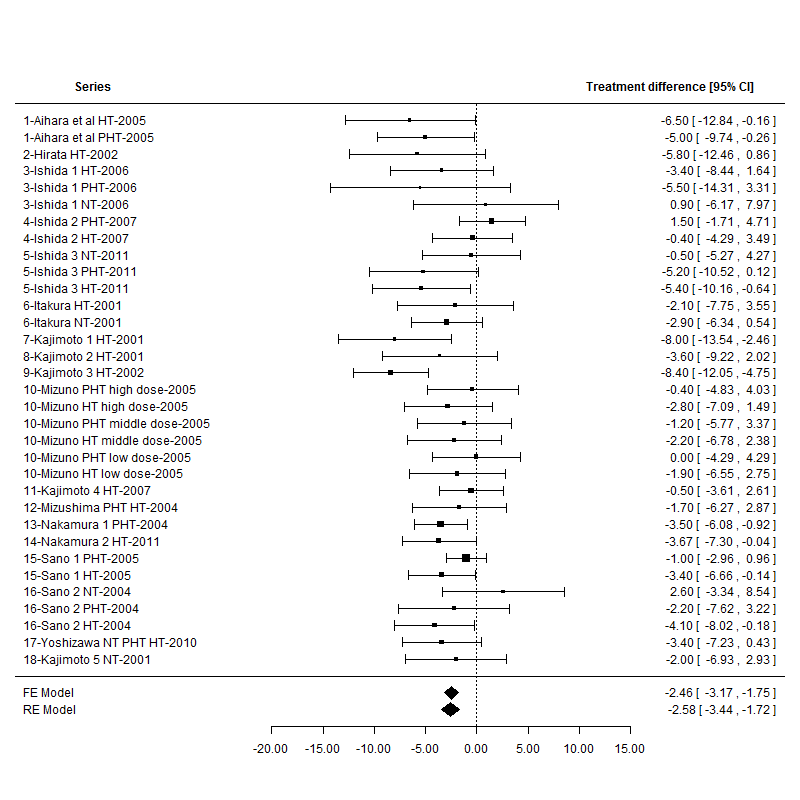

Supplement: S1 Fig — DBP: diastolic blood pressure. FE: fixed effect. HT: hypertensive. NT: normotensive. PHT: pre-hypertensive. RE: random effect. (TIFF) [file pone.0142235.s002.tiff]

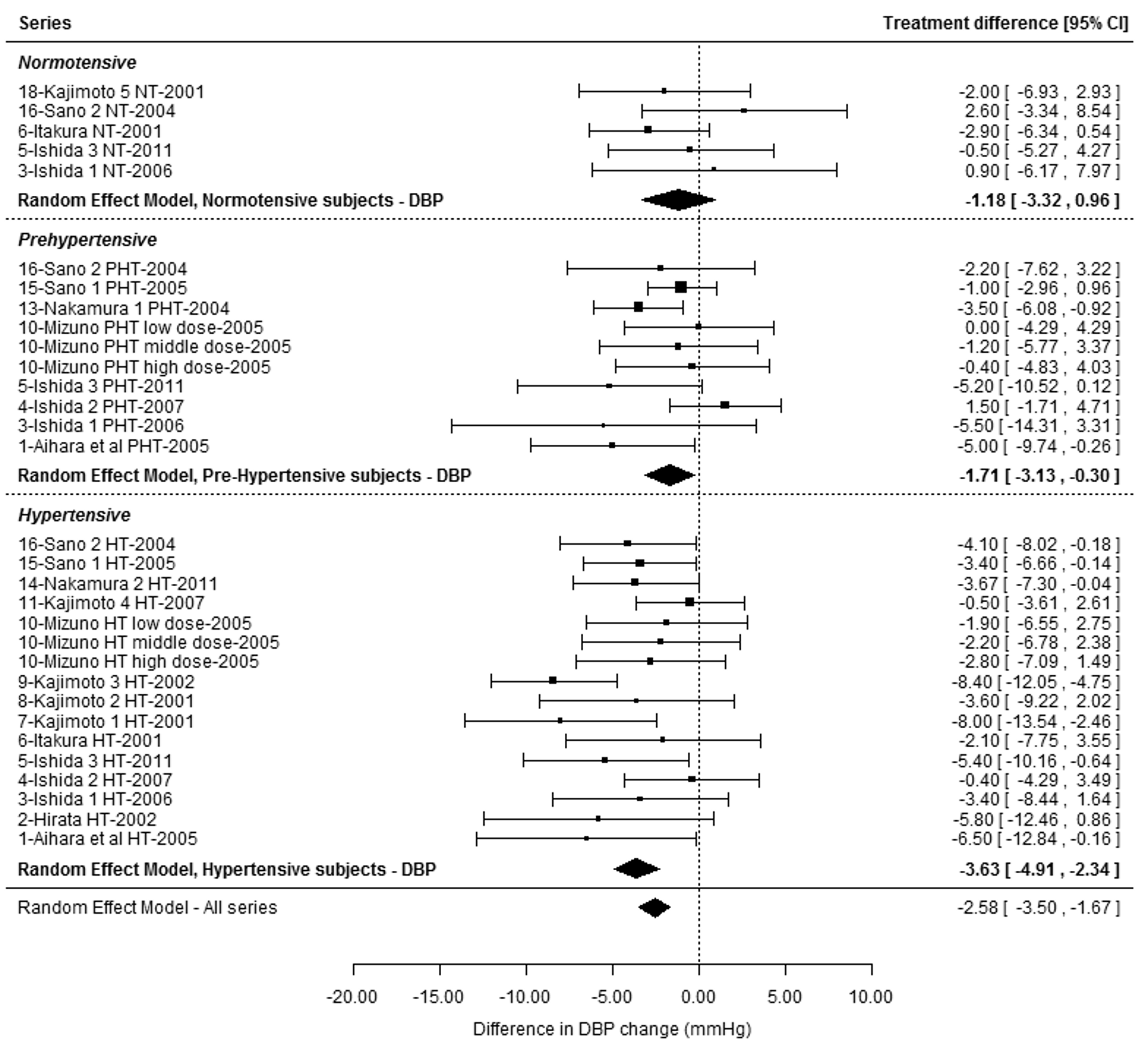

Supplement: S2 Fig — Data on SBP changes were available separately for NT, PHT and/or HT subjects for all studies except two, which were therefore excluded from this subgroup analysis (Mizushima et al 2004 [33] and Yoshizawa et al 2010 [38]). DBP: diastolic blood pressure. HT: hypertensive. NT: normotensive. PHT: pre-hypertensive. (TIFF) [file pone.0142235.s003.tiff]

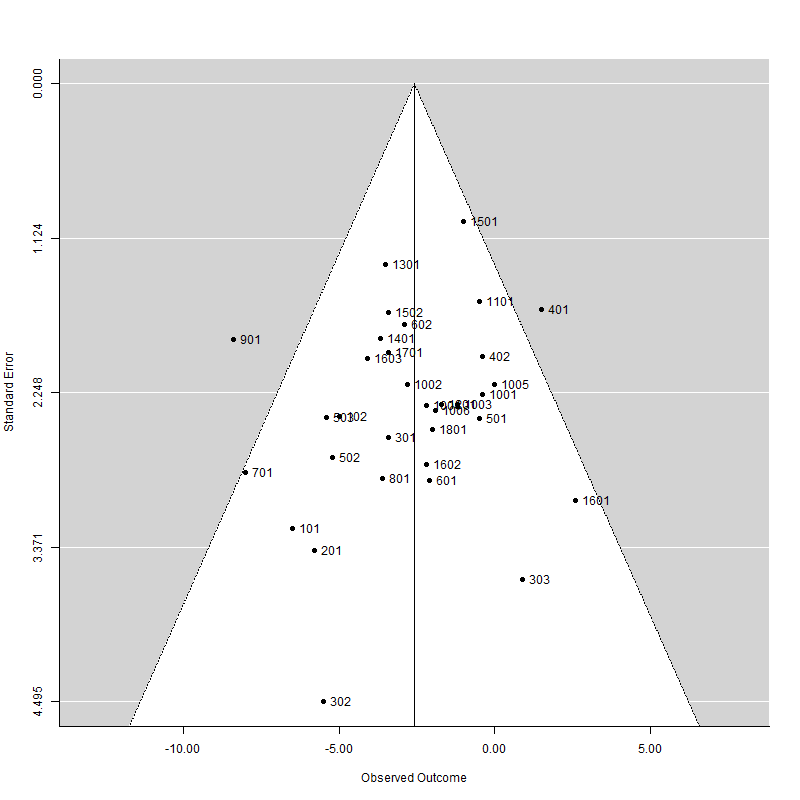

Supplement: S3 Fig — Kendall’s Tau statistic: Kendall’s Tau = -0.1591, P = 0.2001. Series numbers are those indicated in S2 Table. DBP: diastolic blood pressure. IPP: isoleucine–proline–proline. VPP: valine–proline–proline. (TIFF) [file pone.0142235.s004.tiff]
