# Supplementary material for: Influence of the Lactotripeptides Isoleucine–Proline–Proline and Valine–Proline–Proline on Systolic Blood Pressure in Japanese Subjects: A Systematic Review and Meta-Analysis of Randomized Controlled Trials
Source: PLoS One. 2015 Nov 4;10(11):e0142235. doi: 10.1371/journal.pone.0142235 (PMC4633157; doi:10.1371/journal.pone.0142235)
Supplement: S1 Protocol — (PDF) [file pone.0142235.s005.pdf]

Influence of the lactotripeptides Isoleucine-Proline-Proline and Valine-Proline-Proline on blood pressure in Asian subjects: a systematic review and meta-analysis of randomized controlled trials

Aurelie Chanson-Rolle, Veronique Braesco, François Aubin, Toshimitsu Hamasaki, Masafumi Kitakaze

Citation

Aurelie Chanson-Rolle, Veronique Braesco, François Aubin, Toshimitsu Hamasaki, Masafumi Kitakaze. Influence of the lactotripeptides Isoleucine-Proline-Proline and Valine-Proline-Proline on blood pressure in Asian subjects: a systematic review and meta-analysis of randomized controlled trials. PROSPERO 2014:CRD42014014322 Available from http://www.crd.york.ac.uk/PROSPERO_REBRANDING/display_record.asp?ID=CRD42014014322

Review question(s)

Do the lactotripeptides Isoleucine–Proline–Proline and Valine–Proline–Proline reduce systolic blood pressure in Asian subjects ?

What is the influence of the ingested dose of the lactotripeptides Isoleucine–Proline–Proline and Valine–Proline–Proline on the effect on blood pressure?

Searches

The following bibliographic databases had been searched: MEDLINE (via PubMed), Cochrane Central Register of Controlled Trials (via the Cochrane library), J-STAGE (developed by Japan Science and Technology Agency - JST), J Dream III (originally developed by JST and maintained until version II, although transferred to G-search Ltd from version III, Apr 2013). J-STAGE and J Dream III are bibliographic databases for publications written in Japanese language.

MEDLINE and Cochrane were searched until September 17th 2014. J-STAGE was searched until October 1st 2014 and J Dream III was searched until September 25th 2014.

No restrictions (vs language or publication period) were applied to the search.

The following keywords were searched: (lactotripeptide* OR “Dairy peptide*” OR (“Ile-Pro-Pro” AND “Val-Pro-Pro”) OR (“Isoleucyl-prolyl-proline” AND “valyl-prolyl-proline”) OR (“Valine-proline-proline” AND “isoleucine-proline-proline”) OR (“IPP” and “VPP”) OR “Fermented milk” OR “milk fermented” or “sour milk”)

AND

(hypertension OR "blood pressure")

AND

(japan OR japanese OR asian)

The key words "japan OR japanese OR asian" were not used for the search on the Japanese databases.

Paper screening and selection will be performed independently by two team members and discrepancies will be resolved by discussions. All retrieved studies will be screened by the two collaborators on the basis of the reading of titles and abstracts to select studies to be assessed further, and all potentially relevant studies will be further considered by reading the full texts.

Types of study to be included

Inclusion criteria: randomized controlled trials, single blinded or double blinded, involving the consumption of

Isoleucine–Proline–Proline (IPP) and Valine–Proline–Proline (VPP) by Asian adult subjects for longer than 8 days, with office systolic blood pressure measurements at baseline and one/more time points.

Exclusion criteria: reviews (general or systematic reviews, meta-analyses), letters, opinion papers, view points, etc…, non-human studies, studies which are not intervention trials, intervention trials which are not randomized-controlled trials, randomized-controlled trials with an open design, studies in which no measure of SBP was reported, studies in which there was no intervention with IPP+VPP (studies testing IPP alone or VPP alone will be excluded), studies where IPP/VPP intake period is less than 8 days

Condition or domain being studied

Hypertension is a major determinant of health and is likely to have an effect on medical economics worldwide, including in Japan (Nakamura et al 2014). Hypertension can be prevented with lifestyle measures, such as physical activity, maintaining a normal body weight, and adopting a healthy diet. Besides this, several randomized trials and meta-analyses have shown that some peptides derived from milk proteins, such as isoleucine–proline–proline (IPP) and valine–proline–proline (VPP), decrease systolic blood pressure (SBP). However, the size of the effect varies among studies, and it seems to be stronger in Asian subjects than in European subjects (Cicero et al 2011). Several randomized trials have been performed in Japanese subjects. No sufficient meta-analysis has been performed to specifically assess the size of the effect in Asian subjects, especially in Japanese subjects, because many studies have been written in Japanese language and because the literature has not been searched for publications in Japanese language.

Participants/ population

Inclusion: Asian adults (subjects older than 20 yo).

Exclusion: studies in diseased subjects, studies in hypertensive patients receiving blood pressure lowering drugs, studies in children (younger than 20 yo)

Intervention(s), exposure(s)

Intervention to be reviewed will be consumption of the lactotripeptides Isoleucine–Proline–Proline (IPP) and Valine–Proline–Proline (VPP) during more than 8 days

Comparator(s)/ control

Comparison will be made with placebo

Context

Excluded studies: studies with missing information (e.g., change from baseline to endpoint in office SBP) for which no appropriate answer is received from the authors and for which the missing information cannot be calculated/extrapolated from the publication; redundant studies

Outcome(s)

Primary outcomes

Change from baseline to endpoint in systolic blood pressure.

Secondary outcomes

Change from baseline to endpoint in diastolic blood pressure.

Data extraction, (selection and coding)

For studies that fulfill the inclusion criteria, two review team members will independently extract relevant information from the full texts of the selected studies, using a standardized, pre-piloted form. Extracted information:

1. General information: authors, article title, year of publication and references.

2. Study characteristics: country, design, randomization, blinding, duration and dose of IPP/VPP administration, method used to measure blood pressure (e.g., office measurement).

3. Characteristics of participants: age, blood pressure status (normotensive, prehypertensive, hypertensive).

4. Outcomes: primary outcome measure, secondary outcome measure, number of subjects for which study data were analyzed, mean effect and variability measures (SD or SEM or 95% CI).

Delicate issues will be resolved through discussions. Missing data will be requested from study authors.

Risk of bias (quality) assessment
Two team members will be involved in study selection and data extraction, and disagreements will be resolved through discussions. Estimation of the quality of individual studies will be assessed by using the Jadad score. Publication bias will be assessed by means of a funnel plot and asymmetry test (Kendall's tau).

Strategy for data synthesis
Preplanned analyses will be described in a statistical analysis plan. However, the planned general approach for data synthesis will be as follows:

The outcome measure will be mean difference between groups receiving IPP/VPP and those receiving placebo in the change from baseline to endpoint office SBP (primary outcome).

The mean pooled effect of IPP/VPP and its 95% confidence interval will be calculated by using both fixed and random-effect models. Between study heterogeneity will be explored.

The random-effect model meta-analysis will be considered as the primary analysis, on the basis of evidence of heterogeneity between treatment effects in the literature and previous demonstration of significant heterogeneity (Cicero et al 2011).

Analysis of subgroups or subsets
If relevant and if the necessary data are available, we will conduct exploratory analyses to study the influence of relevant covariates. Meta-regressions and sub-group analyses will be used to this end.

Potential covariates will be chosen among: blood pressure status at baseline (normotensive, pre-hypertensive or hypertensive), age, dose of IPP+VPP, duration of IPP+VPP administration.

Dissemination plans
Paper to be submitted to peer-reviewed journal in the relevant field.

Contact details for further information
Dr Chanson-Rolle

aurelie.chanson-rolle@vab-nutrition.com

Organisational affiliation of the review
None

Review team
Dr Aurelie Chanson-Rolle, VAB-nutrition, 1 rue Claude Danziger, 63100 Clermont-Ferrand, France
Dr Veronique Braesco, VAB-nutrition, 1 rue Claude Danziger, 63100 Clermont-Ferrand, France
Dr François Aubin, Venn Life Sciences, 91 Avenue de la République, F-75011 Paris, France
Dr Toshimitsu Hamasaki, Department of Advanced Medical Technology, National Cerebral and Cardiovascular Center, Suita 565-8565, Osaka, Japan
Dr Masafumi Kitakaze, Department of Clinical Medicine and Development, National Cerebral and Cardiovascular Center, Suita 565-8565, Osaka, Japan

Anticipated or actual start date
11 September 2014

Anticipated completion date
31 December 2014

Funding sources/sponsors

The review is sponsored by Calpis Co., Ltd., 11-10, 5-chome, Fuchinobe, Chuo-ku, Sagamihara-shi, Kanagawa 252-0206, Japan

Conflicts of interest

Veronique Braesco, Aurelie Chanson-Rolle and François Aubin have received fees from Calpis for performing the systematic review.

Masafumi Kitakaze received a grant to perform animal and cellular experiments with lactotripeptides under a collaborative research agreement with Calpis.

Toshimitsu Hamasaki has no conflicts of interest in this work.

Language

English, Japanese

Country

France, Japan

Subject index terms status

Subject indexing assigned by CRD

Subject index terms

Asian Continental Ancestry Group; Blood Pressure; Humans; Oligopeptides

Stage of review

Ongoing

Date of registration in PROSPERO

17 October 2014

Date of publication of this revision

30 October 2014

Stage of review at time of this submission	Started	Completed
Preliminary searches	Yes	Yes
Piloting of the study selection process	Yes	Yes
Formal screening of search results against eligibility criteria	Yes	No
Data extraction	No	No
Risk of bias (quality) assessment	No	No
Data analysis	No	No

PROSPERO
International prospective register of systematic reviews
The information in this record has been provided by the named contact for this review. CRD has accepted this information in good faith and registered the review in PROSPERO. CRD bears no responsibility or liability for the content of this registration record, any associated files or external websites.
