# Supplementary material for: Influence of the Lactotripeptides Isoleucine–Proline–Proline and Valine–Proline–Proline on Systolic Blood Pressure in Japanese Subjects: A Systematic Review and Meta-Analysis of Randomized Controlled Trials
Source: PLoS One. 2015 Nov 4;10(11):e0142235. doi: 10.1371/journal.pone.0142235 (PMC4633157; doi:10.1371/journal.pone.0142235)
Supplement: S2 Table — BP: blood pressure. DBP: diastolic blood pressure. IPP: isoleucine–proline–proline. HT: hypertensive. n: number of subjects. NA: not available. NT: normotensive. PHT: pre-hypertensive. VPP: valine–proline–proline. (DOCX) [file pone.0142235.s007.docx]

| Study # | Series number | Study reference | Type of subjects (BP status) | IPP/VPP dose (mg/d) |  | Treated group |  |  | Placebo group |  | Effect size |  |
| --- | --- | --- | --- | --- | --- | --- | --- | --- | --- | --- | --- | --- |
|  |  |  |  |  | n | Change in DBP (mmHg) |  | n | Change in DBP (mmHg) |  | **Mean difference between groups** | **SE** |
|  |  |  |  |  |  | mean | SD |  | mean | SD |  |  |
| 1 | 101 | Aihara 2005 [22] | HT | 13.0 | 20 | NA | NA | 20 | NA | NA | -6.5 | 3.2 |
| 1 | 102 | Aihara 2005 [22] | PHT | 13.0 | 20 | NA | NA | 20 | NA | NA | -5.0 | 2.4 |
| 2 | 201 | Hirata 2002 [39] | HT | 4.3 | 16 | -8.1 | 10.0 | 16 | -2.3 | 9.2 | -5.8 | 3.4 |
| 3 | 301 | Ishida 1-2006 [23] | HT | 15.7 | 9 | 0.3 | 5.3 | 9 | 3.7 | 5.6 | -3.4 | 2.6 |
| 3 | 302 | Ishida 1-2006 [23] | PHT | 15.7 | 9 | -5.8 | 5.3 | 9 | -0.3 | 12.4 | -5.5 | 4.5 |
| 3 | 303 | Ishida 1-2006 [23] | NT | 15.7 | 9 | 2.6 | 8.8 | 9 | 1.7 | 6.3 | 0.9 | 3.6 |
| 4 | 401 | Ishida 2-2007 [24] | PHT | 3.6 | 35 | -1.3 | 6.8 | 36 | -2.8 | 7.0 | 1.5 | 1.6 |
| 4 | 402 | Ishida 2-2007 [24] | HT | 3.6 | 20 | -4.8 | 5.2 | 20 | -4.4 | 7.2 | -0.4 | 2.0 |
| 5 | 501 | Ishida 3-2011 [25] | NT | 17.1 | 8 | -0.1 | 5.3 | 8 | 0.4 | 4.4 | -0.5 | 2.4 |
| 5 | 502 | Ishida 3-2011 [25] | PHT | 17.1 | 8 | -4.1 | 6.0 | 8 | 1.1 | 4.8 | -5.2 | 2.7 |
| 5 | 503 | Ishida 3-2011 [25] | HT | 17.1 | 8 | -7.2 | 4.7 | 8 | -1.8 | 5.0 | -5.4 | 2.4 |
| 6 | 601 | Itakura 2001 [26] | HT | 2.6 | 9 | -5.4 | 8.0 | 9 | -3.3 | 3.3 | -2.1 | 2.9 |
| 6 | 602 | Itakura 2001 [26] | NT | 2.6 | 13 | -2.5 | 4.0 | 13 | 0.4 | 4.9 | -2.9 | 1.8 |
| 7 | 701 | Kajimoto 1-2001 [31] | HT | 4.1 | 15 | -7.4 | 8.5 | 15 | 0.6 | 6.9 | -8.0 | 2.8 |
| 8 | 801 | Kajimoto 2-2001 [27] | HT | 4.2 | 42 | -8.1 | 12.7 | 39 | -4.5 | 13.1 | -3.6 | 2.9 |
| 9 | 901 | Kajimoto 3-2002 [30] | HT | 3.8 | 31 | -9.1 | 7.5 | 33 | -0.7 | 7.4 | -8.4 | 1.9 |
| 10 | 1001 | Mizuno 2005 [32] | PHT | 3.6 | 12 | -1.3 | 6.4 | 12 | -0.9 | 4.5 | -0.4 | 2.3 |
| 10 | 1002 | Mizuno 2005 [32] | HT | 3.6 | 21 | -4.2 | 7.1 | 20 | -1.4 | 6.9 | -2.8 | 2.2 |
| 10 | 1003 | Mizuno 2005 [32] | PHT | 2.5 | 12 | -2.1 | 6.7 | 12 | -0.9 | 4.5 | -1.2 | 2.3 |
| 10 | 1004 | Mizuno 2005 [32] | HT | 2.5 | 21 | -3.6 | 8.0 | 20 | -1.4 | 6.9 | -2.2 | 2.3 |
| 10 | 1005 | Mizuno 2005 [32] | PHT | 1.8 | 12 | -0.9 | 6.1 | 12 | -0.9 | 4.5 | 0.0 | 2.2 |
| 10 | 1006 | Mizuno 2005 [32] | HT | 1.8 | 21 | -3.3 | 8.2 | 20 | -1.4 | 6.9 | -1.9 | 2.4 |
| 11 | 1101 | Kajimoto 4-2007 [29] | HT | 3.6 | 25 | -2.2 | 5.3 | 24 | -1.7 | 5.8 | -0.5 | 1.6 |
| 12 | 1201 | Mizushima 2004 [33] | PHT & HT | 3.1 | 22 | -2.0 | 7.6 | 20 | -0.3 | 7.5 | -1.7 | 2.3 |
| 13 | 1301 | Nakamura 1-2004 [35] | PHT | 3.7 | 53 | -3.8 | 6.3 | 53 | -0.3 | 7.2 | -3.5 | 1.3 |
| 14 | 1401 | Nakamura 2-2011 [34] | HT | 3.4 | 35 | -4.7 | 8.7 | 35 | -1.0 | 6.7 | -3.7 | 1.9 |
| 15 | 1501 | Sano 1-2005 [337] | PHT | 3.1 | 52 | -2.4 | 5.0 | 52 | -1.4 | 5.2 | -1.0 | 1.0 |
| 15 | 1502 | Sano 1-2005 [37] | HT | 3.1 | 20 | -5.2 | 5.1 | 20 | -1.8 | 5.4 | -3.4 | 1.7 |
| 16 | 1601 | Sano 2-2004 [36] | NT | 9.2 | 6 | -1.7 | 5.5 | 5 | -4.3 | 4.3 | 2.6 | 3.0 |
| 16 | 1602 | Sano 2-2004 [36] | PHT | 9.2 | 8 | -3.5 | 6.1 | 8 | -1.3 | 4.9 | -2.2 | 2.8 |
| 16 | 1603 | Sano 2-2004 [36] | HT | 9.2 | 8 | -5.8 | 4.0 | 8 | -1.7 | 4.0 | -4.1 | 2.0 |
| 17 | 1701 | Yoshizawa 2010 [38] | NT, PHT & HT | 6.7 | 12 | -2.5 | 4.7 | 10 | 0.9 | 4.4 | -3.4 | 2.0 |
| 18 | 1801 | Kajimoto 5-2001 [28] | NT | 12.4 | 21 | -3.0 | 8.2 | 22 | -1.0 | 8.3 | -2.0 | 2.5 |
